# Efficacy and Safety of Traditional Chinese Medicine in Idiopathic Pulmonary Fibrosis: A Meta-Analysis

**DOI:** 10.1155/2020/1752387

**Published:** 2020-02-13

**Authors:** Kun Ji, Jianling Ma, Liangmin Wang, Niuniu Li, Shangjuan Dong, Liqing Shi

**Affiliations:** Department of Respiratory Medicine, Dongfang Hospital, Beijing University of Traditional Chinese Medicine, Beijing 100078, China

## Abstract

**Objective:**

To evaluate the efficacy and safety of traditional Chinese medicine (TCM) on lung function and quality of life of idiopathic pulmonary fibrosis (IPF) patients by meta-analysis.

**Methods:**

Randomized controlled trials (RCTs) related to TCM and IPF were searched on PubMed, EMBASE Cochrane Library, ClinicalTrials, China National Knowledge Infrastructure (CNKI), Wanfang Database, Chin VIP Information (VIP), and Chinese Biomedical Database (CBM) until December 2018. Standard mean difference (SMD) and 95% CI were calculated for the measurements related to lung function (FEV1/FVC, FVC%, FEV1%, TLC%, DLCO% or DLCO, and VC%) and other parameters (PO_2_, 6MWD, and SGRQ) when comparing TCM treatment to the control group. Relative risk (RR) and 95% CI of adverse events (AEs) were calculated to assess the safety of TCM.

**Results:**

A total of 40 RCTs comparing TCM to western medicine (WM) and involving 3194 IPF patients were eligible for the meta-analysis. The pooled results showed that TCM treatment improved significantly PO_2_ (SMD = 0.80, 95% CI 0.54 to 1.06, *p* < 0.001), FEV1% (SMD = 0.57, 95% CI 0.42 to 0.71, *p* < 0.001), DLCO% (SMD = 0.38, 95% CI 0.28 to 0.48, *p* < 0.001), 6MWD (SMD = 0.70, 95% CI 0.56 to 0.84, *p* < 0.001) and other measurements and reduced SGRQ scores (SMD = −0.51, 95% CI −0.70 to −0.22, *p* < 0.001). Subgroup analysis of different study durations (3 months, ≥ 6 months) and comparison models (TCM vs. WM, TCM + WM vs. WM or TCM vs. placebo) showed similar results. No significant difference of risk of AEs was observed between both groups (RR = 0.66, 95% CI: 0.27–1.60, *p*=0.352). There was no obvious publication bias, and the pooled results were stable according to sensitivity analysis.

**Conclusion:**

To the best of our knowledge, the present study had the largest sample size. Our results indicated that TCM treatment may help provide benefit to the lung function, exercise capacity, and quality of life of IPF patients, alone or combined with WM, when compared to WM. More rigorous RCTs were needed in the future.

## 1. Introduction

Idiopathic pulmonary fibrosis (IPF) is a rare disease characterized by chronic, progressive, and fibrosing interstitial pneumonia with undetermined etiology. It is the most common type of idiopathic interstitial pneumonia. The incidence of IPF varies in different populations and has risen over time. It is estimated to range from 2.8 to 18 cases in the western countries and from 0.5 to 4.2 cases in the Asia and South American, per 100000 people per year [1, 2]. Although the exact etiology remains unclear, multiple environmental exposures and genetic factors have been implicated [3].

IPF is a life-threatening disease with extremely poor prognosis. As the disease progresses, the lung function continues to decline despite treatments, which ultimately leads to respiratory failure and even death. The median survival time from diagnosis is only 2 to 3 years [4]. Currently, the pharmacological therapy for IPF includes glucocorticoid, immunosuppressive or cytotoxic agents, tyrosine kinase inhibitor, and antifibrotic agents. Yet, randomized trials have identified some drugs were potentially ineffective or harmful [5, 6], whereas only two, pirfenidone and nintedanib, were proved to be effective disease-modifying therapies for IPF [7, 8]. Besides pharmacological therapy, lung transplantation can prolong survival with a 5-year survival of near 50% and improve quality of life of IPF patients [9]. However, only a few patients can receive transplantation due to the high expense and lack of donors organs. Overall, there is an urgent need to develop new therapy for IPF with clear effect and less adverse events.

In recent years, traditional Chinese medicine (TCM) has been reported to have effect on IPF in animal models and patients [10]. TCM was recommended as an experimental therapy for IPF, and many kinds of TCM were widely clinically used in China. However, due to the lack of large-scale, multicentered, randomized controlled trials, the treatment efficacy of TCM on IPF is still in controversy. In the present study, we aimed to evaluate the efficacy of TCM on IPF by meta-analysis.

## 2. Methods

### 2.1. Literature Search

Literature search was performed on English databases including PubMed, EMBASE, Cochrane Library, and ClinicalTrials, and Chinese databases including China National Knowledge Infrastructure (CNKI), Wanfang Database, Chin VIP Information (VIP) and Chinese Biomedical Database (CBM) until December 2018. Different combinations of the following terms were used: (IPF OR pulmonary fibrosis OR lung fibrosis) AND (Chinese medicine OR herbal medicine OR herbal drug).

### 2.2. Including and Excluding Criteria

Eligible studies should fulfill the following criteria: (1) randomized controlled trials (RCTs) with regards to the efficacy of Tradition Chinese Medicine (TCM) to idiopathic pulmonary fibrosis; (2) comparing TCM vs. placebo or TCM vs. western med (WM) or TCM + WM vs. WM; (3) reporting at least one of the following outcomes with measured values or the percentages to the predicted value: VC (vital capacity), FEV1 (forced expiratory volume in one second), FVC (forced vital capacity), TLC (total lung capacity), FEV1/FVC, DLCO (diffusion capacity of the lung for carbon monoxide), PO_2_ (partial pressure of oxygen), 6MWD (6-minute walking distance), SGRQ scores (St. George's respiratory questionnaire), CAT (COPD assessment test), SF-36 Health Survey questionnaire, ATAQ-IPF (a tool to assess quality of life in idiopathic pulmonary fibrosis), and AEs; (4) lasting at least of 3 months or 12 weeks. Cases reports, reviews, non-RCTs, duplications, and those comparing different TCMs or assessing nondrug treatments (acupuncture and taichi for example) were all discarded.

### 2.3. Quality Assessment

The quality of all eligible studies was assessed according to the Jadad scale comprised of three items: randomization, blinding, withdrawals, and dropouts [11]. Zero, one, or two scores were given according to the description and appropriateness of these items. A study with a total score ≥3 was considered to be of high quality; otherwise, it was of low quality.

### 2.4. Data Extraction

The following information of each eligible study were extracted: the first author, year of publishing, regimens of the treatment group and control group, number and mean age of patients in each group, duration of the trial and the measurements of clinical outcomes. Specifically, the literature search, study filtering, quality assessment, and data extraction were performed by two independent researchers. If disagreements occurred in any step, the researchers would discuss the details until they reached a consensus.

### 2.5. Statistics

The heterogeneity between studies was calculated by *I*^2^ statistics. If there was low heterogeneity (*I*^2^ < 50%), the fixed-effect model would be used. Otherwise, the random-effect model was used. Standard mean difference (SMD) and the 95% CI of each outcome measurement were calculated to assess the difference of treatment efficacy between different regimens. Relative risk (RR) and 95% CI of AEs were calculated to assess the safety of TCM. Additionally, we performed the subgroup analysis according to the trial duration and comparison of regimens as follows:

1. Duration subgroups: 3 months (or 12 weeks); 6 months (or 24 weeks) or more
2. Comparison subgroups: TCM vs. WM; TCM + WM vs. WM or TCM vs. placebo

However, only the subgroups involving at least 3 studies were analyzed. Sensitivity analysis was performed to identify if any study had significant impact on the pooled results of meta-analysis. Egger's test and funnel plot were used to assess the publication bias. All statistics were performed by using STATA 11.0 (StataCorp LP, TX, USA). *p* value less than 0.05 indicated statistical significance.

## 3. Results

### 3.1. Characteristics of All Eligible Studies

**Figure 1:**
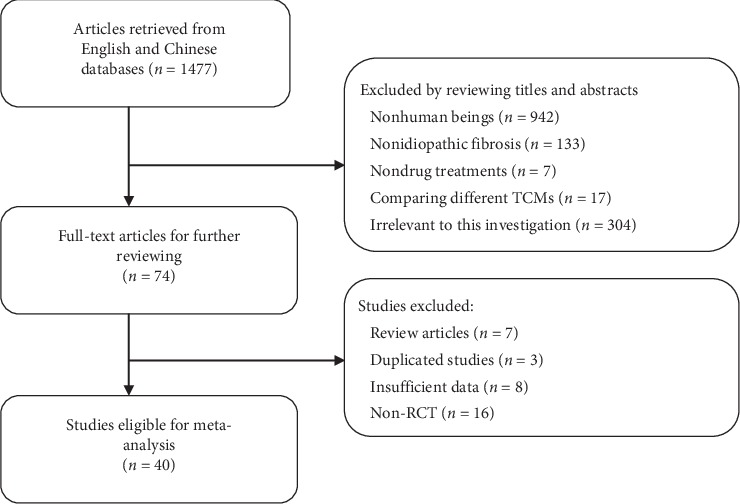
Flowchart of literature reviewing and filtering.

Literature search retrieved a total of 1477 published articles, of which 1403 did not fulfill the inclusion/exclusion criteria and were discarded after screening the titles and abstracts. The remaining articles were reviewed for the full text and 34 articles were subsequently excluded according to the inclusion/exclusion criteria. Finally, 40 studies were included in the present meta-analysis [12–51]. The flowchart of literature review is shown in Figure 1.

A total of 3194 participants were involved in the analysis, of which 1647 were in the intervention group (TCM only or TCM + WM) and 1547 were in the control group (WM or placebo). The sample size of each study ranged from 34 to 324 and the duration varied from 3 months (or 12 weeks) to 18 months. As for the regimen, 29 studies compared the effect of TCM plus WM to WM alone in IPF, of which 21 were TCM + glucocorticoid versus glucocorticoid (prednisone or dexamethasone) [12–14, 17, 18, 20, 21, 23, 24, 29, 31, 32, 35–37, 39, 40, 42, 44, 48, 49], 6 were TCM + N-acetylcysteine versus N-acetylcysteine [26, 28, 33–35, 40], one was TCM + edaravone versus edaravone [30] and one was TCM + pirfenodone versus pirfenodone [37]. The other 11 studies compared TCM versus placebo [43] or TCM versus WM [14, 16, 19, 22, 25, 27, 28, 41, 46, 51]. Additionally, one trial [22] assigned patients to different dosage groups (high or low dosage group), and therefore, each dosage group was separately pooled in the meta-analysis. None of the included studies reported results of CAT, SF-36, and ATAQ-IPF, and these outcomes were not analyzed in the present study. We also assessed the quality of each randomized trial by Jadad scores and found that 5 studies were of high quality (Jadad scores ≥ 3) [22, 23, 43, 48, 51], whereas the rest were of low quality. The characteristic of all included studies are listed in Table 1.

### 3.2. Lung Function

There were 6 and 11 studies evaluating the change of FEV1/FVC or FVC% in both groups, respectively (Table 2). High heterogeneities were found in both analyses (*I*^2^ > 50%) and random-effect model was applied. The pooled results showed significant differences of FEV1/FVC (SMD = 0.90. 95% CI 0.48 to 1.31, *p* < 0.001) and FVC% (SMD = 0.60, 95% CI 0.40 to 0.80, *p* < 0.001) between TCM and control groups.

**Figure 2:**
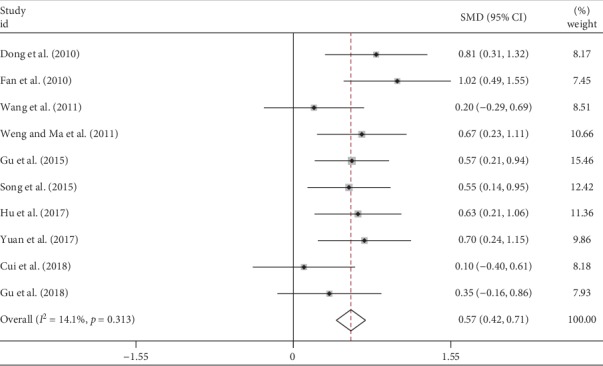
Forest plot of meta-analysis of FEV1% difference between TCM and control groups.

We included 10 studies measuring FEV1% in IPF patients in the meta-analysis with a total of 781 cases (Table 2). There was low heterogeneity between studies (*I*^2^ = 14.1%) so that the fixed-effect model was used. The pooled analysis showed a significant improvement of FEV1% in the TCM group than that in the control group (SMD = 0.57, 95% CI 0.42 to 0.71, *p* < 0.001) as shown in Figure 2.

**Figure 3:**
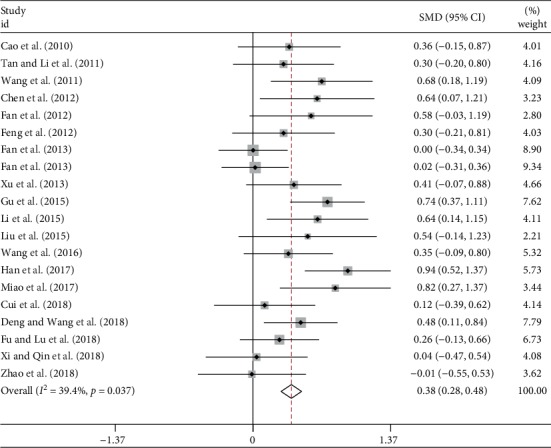
Forest plot of meta-analysis of DLCO% difference between TCM and control groups.

Additionally, 20 studies comprising 1518 patients reported changes of DLCO% before and after intervention in both groups (Table 2), and there was a moderate heterogeneity (*I*^2^ = 39.4%). Pooled analysis (Figure 3) in the fixed-effect model showed TCM treatment significantly improved DCLO% when comparing to the control group (SMD = 0.38, 95% CI 0.28 to 0.48, *p* < 0.001).

We also compared the other measurements related to lung function (FVC%, TLC%, DLCO, VC%) between TCM and control groups by meta-analysis (Table 2). There was no or low heterogeneity, and pooled results indicated significant improvements of these measurements in the TCM group than that in the control group (*p* for SMD <0.001).

### 3.3. Other Parameters

**Figure 4:**
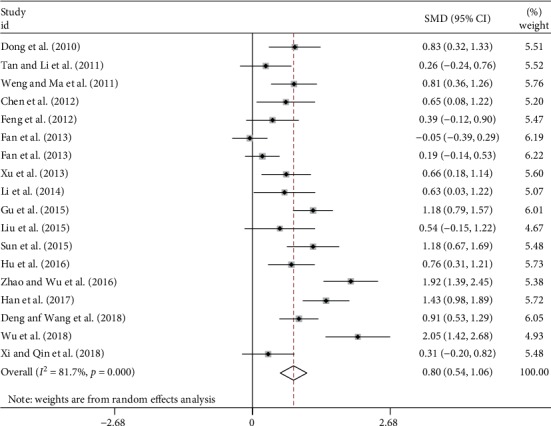
Forest plot of meta-analysis of PO_2_ difference between TCM and control groups.

A total of 18 studies assessed the effect of TCM on PO_2_ change in IPF with 717 patients in the treatment group and 705 in control group (Table 2). There was a high heterogeneity (*I*^2^ = 81.7%) so the random-effect model was used. After pooling analysis, the SMD was 0.80 (95% CI 0.54 to 1.06, *p* < 0.001), indicating that the TCM group had significant improvement of PO_2_ compared to the control group (Figure 4).

**Figure 5:**
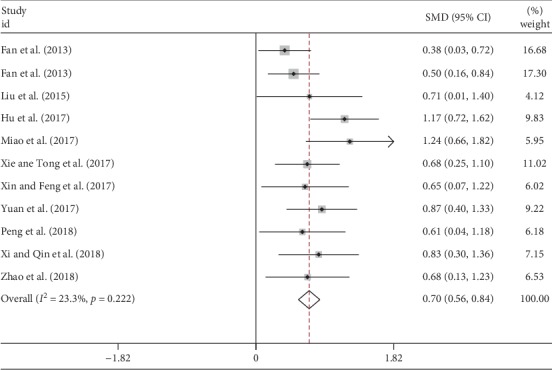
Forest plot of meta-analysis of 6MWD difference between TCM and control groups.

There were 11 studies with 828 IPF patients measuring 6MWD and were included in our analysis (Table 2). Low heterogeneity was found (*I*^2^ = 23.3%) and then the fixed-effect model was applied. The pooled analysis indicated a significant improvement of 6MWD in TCM group compared with the control group (SMD = 0.70, 95% CI 0.56 to 0.84, *p* < 0.001) as shown in Figure 5.

**Figure 6:**
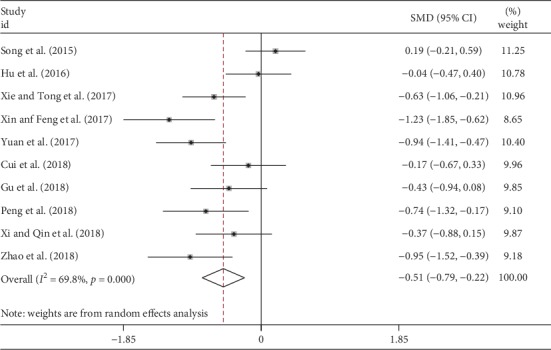
Forest plot of meta-analysis of SGRQ scores difference between TCM and control groups.

SGRQ scores were reported in 10 studies involving 342 patients in the TCM group and 336 patients in the control group (Table 2). The random-effect model was used due to obvious heterogeneity (*I*^2^ = 69.8%). After pooling analysis (Figure 6), TCM treatment significantly decreased SGRQ scores compared with the control group (SMD = −0.51, 95% CI −0.70 to −0.22, *p* < 0.001).

### 3.4. Subgroup Analysis

Subgroup analyses were performed according to study duration, so as to assess the short term (3 months) and long term (6 months or more) effect, and different comparison models (TCM vs. WM, TCM + WM vs. WM or TCM vs. placebo). We only analyzed those subgroups involving at least 3 studies. For the duration subgroups (Table 3), TCM treatment had significant improvement of all measurements relating to lung function or life quality when compared with the control group in either the short-term subgroup or the long-term subgroup. In the TCM + WM vs. WM or TCM vs. placebo subgroup (Table 4), all measurements were significantly improved in TCM group. In the TCM vs. WM subgroup, similar results of each measurement were seen except PO_2_ and VC% which showed no significant difference between both groups.

### 3.5. Safety

Only 14 studies reported the outcomes of adverse events (AEs), of which 6 observed no AEs and 8 reported various AEs, including stomach upset, nausea, dizziness, constipation, insomnia, slightly elevated blood glucose, and so on. Since the AE profiles varied among studies, only overall prevalence of AEs was compared between treatment and control groups. In the treatment group, 20 (3.4%, 20/591) AEs were observed while 35 (7.2%, 35/487) AEs were observed in the control group. In the two studies [16, 22], the TCM group had lower risk of AEs than the control group (RR = 0.04 (95% CI: 0.00–0.63) and RR = 0.11 (95% CI: 0.02–0.51), respectively). However, after pooled analysis of all studies using random-effect model, there was no significant difference of AEs between both groups (RR = 0.66, 95% CI: 0.27–1.60, *p*=0.352, *I*^2^ = 51.8%), indicating that TCM is well-tolerated.

### 3.6. Sensitivity Analysis and Publication Bias

We performed sensitivity analysis in each meta-analysis and found no single study could significantly affect the pooled results, indicating a high stability of our analysis. The funnel plots were symmetric and Egger's tests suggested no obvious publication bias.

## 4. Discussions

In recent decades, repetitive microinjuries to epithelium caused by the interaction of various environmental and genetic risk factors have been thought to play crucial role in the development of IPF [3]. These microinjuries cause aberrant communication between epithelium and fibroblast, the production of myofibroblasts, accumulation of extracellular matrix, and finally remodel lung interstitium [3]. With the development of the extraction and purification of herbs, TCM has been proved to be effective on IPF in animal models and exert some effect against the pathophysiology of IPF mentioned above [10]. These TCMs were found to inhibit the proliferation of fibroblast cells, downregulate the expression of matrix-related genes and induce the apoptosis of abnormal lung fibroblast [52, 53]. However, the clinical effect of TCM on IPF was controversial in spite of its wide usage in China.

In present study, we systematically reviewed and pooled the results of 40 studies comprising 3194 individuals to comprehensively evaluate the efficacy of TCM on the improvement of lung function and life quality of IPF. We found that TCM had significant impact on the change of lung function, exercise capacity, and life quality before and after treatment. After pooling analysis, there was significant difference between the change of PO_2_, FEV1/FVC, FVC%, FEV1%, TLC%, DLCO% or DLCO, and VC% in the TCM and control groups. Similar results were observed when TCM was used alone or in combination with western medicines, indicating TCM may help improve lung function or slow the decline of lung function of IPF. On the other side, there was significant improvement of 6MWD and decrease of SGRQ scores in the TCM treatment group compared with the control group, suggesting that TCM can improve the exercise capacity and quality of life of IPF patients. In addition, we found similar risk of adverse events between TCM and control groups, indicating that TCM is safe for IPF patients. There was no obvious heterogeneity and publication bias in most of the pooling analyses. Sensitivity analysis suggested that these pooled results were stable.

However, there were some limitations in the present study. Firstly, the quality of the majority of included studies was poor because the blindness was unclear and dropouts were not mentioned. More well-designed, double-blinded, multicentered RCTs in high quality are needed. Secondly, we pooled all TCMs together in the present study instead of analyzing one single TCM compounds due to limited number of studies related to each compound. TCM compounds are complicated and may have various effects on IPF. The present study may over- or underestimate the effect of some certain compounds. Meta-analysis aiming to precisely evaluate the efficacy of one single TCM compound on IPF is necessary. Thirdly, only a small proportion of studies (35%) reported the outcomes of adverse events and the safety of TCM needs to be confirmed by more studies. Fourthly, moderate-to-high heterogeneity existed in most of the analyses and the interpretation of our results needs caution. Finally, the quality of life was only assessed using SGRQ scores. The other measurements that may be more appropriate, including CAT, SF-36, and ATAQ-IPF, were not analyzed due to insufficient data. In the future, more studies using these tools may be needed.

## 5. Conclusions

In conclusion, we performed a meta-analysis involving 40 RCTs, which may have the largest sample size as far as we know, and found that TCM treatment may help improve the lung function, exercise capacity, and quality of life in IPF patients and the treatment is safe. However, more randomizes controlled clinical trials are needed for in the future.

## Figures and Tables

**Table 1:** Characteristics of all studies included in the meta-analysis.

Study	Regimen		No. of patients^*∗*^	Age (years)^*∗*^	Duration^#^	Outcomes	Jadad
	Treatment	Control					
Cao et al. [12]	Tongluo Huaxian granules + PDN	PDN	30/30	60.27/61.54	6 M	VC%, TLC%	2
Dong [13]	Kangxian Shufei granules + PDN	PDN	33/33	59.11/57.7	3 M	DLCO%, PO_2_, FEV1%, FVC%	1
Fan et al. [14]	Kechuanting granules	PDN	32/31	54.11/53.03	6 M	FEV1%, FEV1/FVC,	1
Tan and Li [15]	Shuizhi Tongluo capsule + PDN	PDN	31/31	61.1/63.67	3 M	DLCO%, PO_2_	1
Wang et al. [16]	Yangyin Yiqi Misture	PDN	34/30	66.07/63.13	6 M	DLCO%, FEV1%, FVC%,	2
Weng and Ma [17]	Qingjin decoction + PDN	PDN	42/42	52.66/53.33	6 M	PO_2_, FEV1%,	2
Chen et al. [18]	Huaxian Pogu formula + PDN	PDN	25/25	63/65	3 M	VC%, DLCO%, TLC%, PO_2_	1
Fan et al. [19]	Feixiantong decoction	NAC	22/21	60.98/65.12	12 W	VC%, DLCO%, TLC%	2
Feng et al. [20]	Feixiankang granules + PDN	PDN	30/30	—	6 M	DLCO%, PO_2_	1
Gan et al. [21]	Huaxian decoction + PDN	PDN	26/27	63.2/64.5	3 M	DLCO	2
Fan et al. [22]	Feitong oral liquid (high dose)	PDN	73/65	59.01/58.27	3 M	DLCO%, TLC%, PO_2_, 6MWD	5
Fan et al. [22]	Feitong oral liquid (low dose)	PDN	66/65	57.38/58.27	3 M	DLCO%, TLC%, PO_2_, 6MWD	5
Xu et al. [23]	Bufei Yishen Huoxue decoction + PDN	PDN	35/35	52.32/51.51	12 W	DLCO%, PO_2_, FVC%,	3
Chen [24]	Danhong injection + PDN	PDN	45/45	50.2/49.5	12 W	DLCO	2
Li et al. [25]	Feibitongfang	PDN	23/22	59.97/64.59	12 W	VC%, TLC%, PO_2_	2
Yan et al. [26]	Buyang Huanwu decoction + NAC	NAC	60/60	63.5/63.4	6 M	DLCO%, PO_2_, FEV1%	2
Li et al. [27]	Yangyin Yifei Tongluo Wan	NAC	34/30	58.23/59.98	3 M	VC%, DLCO%, TLC%	2
Liu et al. [28]	Xuefu Zhuyu capsule + NAC	NAC	18/16	—	18 M	DLCO%, TLC%, PO_2_, FVC%, 6MWD	2
Song [29]	Xuefu Zhuyu decoction + PDN	PDN	48/48	62.1/63.27	3 M	FEV1%, FVC%, SGRQ	2
Sun et al. [30]	Danhong injection + ED	ED	35/35	48.2/50.8	12 W	DLCO, PO_2_	2
Yan [31]	Danhong injection + PDN	PDN	34/34	52.3/53.5	12 W	DLCO	1
Hu et al. [32]	Yiqi Huayu Tongluo decoction + PDN	PDN	40/40	53.87/54.19	12 W	DLCO, PO_2_, SGRQ	2
Wang et al. [33]	Wenyang Huayu decoction + NAC	NAC	43/37	—	3 M	VC%, DLCO%, TLC%,	1
Zhao and Wu [34]	Danhong Injection + NAC	NAC	40/40	62.3/62.8	12 W	DLCO, PO_2_, FEV1/FVC	1
Han et al. [35]	Pingfeng Shengmai powder + PDN	PDN	47/47	59.23/58.64	3 M	VC%, DLCO%, TLC%, PO_2_	1
Hu [36]	Pingfeng Shengmai powder + PDN	PDN	45/44	56.21/56.68	6 M	FEV1%, FVC%, 6MWD	1
Li et al. [37]	Peiyuan Huoxue decoction + PDN	PDN	42/42	59.4/59.8	6 M	FEV1/FVC	1
Miao et al. [38]	Bushen Tongluo decoction	NAC	28/27	62.36/65.68	12 W	VC%, DLCO%, 6MWD, SGRQ	2
Wang [39]	Xuefu Zhuyu decoction + PDN	PDN	162/162	60.6/61.3	24 W	FVC%	2
Xie and Tong [40]	Yangyin Yiqi Misture + DXM	DXM	45/45	—	3 M	FEV1/FVC, 6MWD, SGRQ	2
Xin and Feng [41]	Erjiaxiaozheng decoction	NAC	25/24	65.68/65.88	6 M	6MWD, SGRQ	1
Yuan et al. [42]	Bufei Yishen Huoxue Misture + DXM	DXM	39/39	68.56/69.61	3 M	FEV1%, FEV1/FVC, 6MWD, SGRQ	2
Cui et.al. [43]	Fuzheng Tixie Souluo decoction	Placebo	30/31	63.9/62.61	12 W	VC%, DLCO%, TLC%, FEV1%, FVC%, SGRQ	4
Deng and Wang [44]	Huangqi Taohong decoction + PDN	PDN	59/59	64.06/63.21	3 M	DLCO%, PO_2_, FVC%	2
Feng and Sun [45]	Yifei Tongluo recipe + NAC	NAC	31/31	61/62.3	6 M	DLCO, 6MWD	2
Fu and Lu [46]	Loubei Lengshu decoction	PDN	50/50	—	3 M	DLCO%, TLC%	2
Gu [47]	Qishufeixian decoction + PFD	PFD	30/30	62.3/63.8	6 M	FEV1%, FEV1/FVC, FVC%, SGRQ	2
Peng et al. [48]	Qigui recipe + PDN	PDN	25/25	58.96/59.8	12 W	6MWD, SGRQ	3
Wu [49]	Danhong injection + PDN	PDN	30/30	61.56/61.8	12 W	DLCO, PO_2_	2
Xi and Qin [50]	Huaxian Tongluo decoction + NAC	NAC	30/30	65.11/64.28	3 M	VC%, DLCO%, TLC%, PO_2_, 6WMD, SGRQ	2
Zhao et al. [51]	Fuzheng Huaxian formula	NAC	30/24	58/59	3 M	DLCO%, FVC%, 6MWD, SGRQ	3

^*∗*^Treatment/control; ^#^M = months, W = weeks; 6MWD: 6-minute walking distance; SGRQ: St. George's respiratory questionnaire; VC: vital capacity; FEV1: forced expiratory volume in one second; TLC: total lung capacity; FVC: forced vital capacity; DLCO: diffusion capacity of the lung for carbon monoxide; NAC: N-acetylcysteine; PDN: prednisone; DXM: dexamethasone; PFD: pirfenidone; ED: edaravone; % indicates the percentage of measured value to predicted value.

**Table 2:** Meta-analysis of the efficacy of TCM on idiopathic pulmonary fibrosis.

Measurements	No. of studies	No. of patients	Effect size			*I* ^2^ (%)
			SMD	95% CI	*p*	
PO_2_	18	717/705	0.80	0.54, 1.06	<0.001	81.7
FEV1/FVC	6	228/227	0.90	0.48, 1.31	<0.001	77.7
FVC%	11	524/512	0.60	0.40, 0.80	<0.001	56.2
FEV1%	10	393/388	0.57	0.42, 0.71	<0.001	14.1
TLC%	13	491/469	0.27	0.15, 0.40	<0.001	0
DLCO%	20	775/743	0.38	0.28, 0.48	<0.001	39.4
DLCO	8	281/282	1.19	1.01, 1.37	<0.001	45.6
VC%	10	312/300	0.35	0.19, 0.51	<0.001	30.4
6MWD	11	424/404	0.70	0.56, 0.84	<0.001	23.3
SGRQ	10	342/336	−0.51	−0.70, −0.22	<0.001	69.8

TCM: traditional Chinese medicine; SMD: standard mean difference.

**Table 3:** Subgroup analysis of the efficacy of TCM on idiopathic pulmonary fibrosis according to study duration^*∗*^.

Measurements	3 months			6 months or more		
	SMD (95% CI)	*p*	*I* ^2^ (%)	SMD (95% CI)	*p*	*I* ^2^ (%)
PO_2_	0.82 (0.50, 1.14)	<0.001	84.8	0.77 (0.41, 1.14)	<0.001	55.0
FEV1/FVC	1.31 (1.04, 1.59)	<0.001	45.8	0.50 (0.22, 0.78)	<0.001	15.1
FVC%	0.50 (0.15, 0.86)	0.005	71.3	0.75 (0.58, 0.92)	<0.001	0
FEV1%	0.55 (0.32, 0.79)	<0.001	33.2	0.58 (0.39, 0.76)	<0.001	15.9
TLC%	0.25 (0.12, 0.38)	<0.001	0	—	—	—
DLCO%	0.33 (0.22, 0.45)	<0.001	44.8	0.56 (0.34, 0.78)	<0.001	0
DLCO	1.23 (1.04, 1.42)	<0.001	48.8	—	—	—
VC%	0.34 (0.17, 0.50)	<0.001	36.9	—	—	—
6MWD	0.65 (0.49, 0.81)	<0.001	17.0	0.92 (0.60, 1.23)	<0.001	16.3
SGRQ	−0.44 (−0.74, −0.13)	0.005	69.7	—	—	—

^*∗*^Only the subgroups involving at least 3 studies were analyzed; TCM: traditional Chinese medicine.

**Table 4:** Subgroup analysis of the efficacy of TCM on idiopathic pulmonary fibrosis according to comparison models^*∗*^.

Measurements	TCM vs. WM			TCM + WM vs. WM or TCM vs. Placebo		
	SMD (95% CI)	*p*	*I* ^2^ (%)	SMD (95% CI)	*p*	*I* ^2^ (%)
PO_2_	0.35 (−0.03, 0.73)	0.072	68.7	0.93 (0.66, 1.20)	<0.001	75.9
FEV1/FVC	—	—	—	0.98 (0.52, 1.44)	<0.001	78.7
FVC%	0.52 (0.23, 0.82)	0.001	38.5	0.62 (0.38, 0.87)	<0.001	62.8
FEV1%	0.67 (0.19, 1.16)	0.007	63.6	0.53 (0.37, 0.70)	<0.001	0
TLC%	0.19 (0.02, 0.36)	0.028	0	0.37 (0.18, 0.56)	<0.001	24.4
DLCO%	0.33 (0.10, 0.57)	0.006	53.5	0.46 (0.33, 0.60)	<0.001	16.8
DLCO	1.19 (1.01, 1.37)	<0.001	45.6	—	—	—
VC%	0.39 (0.00, 0.79)	0.050	50.6	0.33 (0.14, 0.53)	0.001	26.5
6MWD	0.58 (0.39, 0.78)	<0.001	40.0	0.83 (0.63, 1.04)	<0.001	0
SGRQ	—	—	—	−0.38 (−0.65, −0.10)	0.008	63.5

^*∗*^Only the subgroups involving at least 3 studies were analyzed; TCM: traditional Chinese medicine; WM: western medicine.

## Data Availability

The data used to support the findings of this study are included within the article.
